# Genetic Divergence and Dispersal of Yellow Fever Virus, Brazil

**DOI:** 10.3201/eid1009.040197

**Published:** 2004-09

**Authors:** Pedro F.C. Vasconcelos, Juliet E. Bryant, Amelia P.A. Travassos da Rosa, Robert B. Tesh, Sueli G. Rodrigues, Alan D.T. Barrett

**Affiliations:** * World Health Organization Collaborating Center for Arbovirus Reference and Research, Instituto Evandro Chagas, Belém, Brazil;; † University of Texas Medical Branch, Galveston, Texas, USA

**Keywords:** yellow fever virus, Brazil, evolution, epidemiology, enzootic, 3′NCR, genetic divergence, research

## Abstract

An analysis of 79 yellow fever virus (YFV) isolates collected from 1935 to 2001 in Brazil showed a single genotype (South America I) circulating in the country, with the exception of a single strain from Rondônia, which represented South America genotype II. Brazilian YFV strains have diverged into two clades; an older clade appears to have become extinct and another has become the dominant lineage in recent years. Pairwise nucleotide diversity between strains ranged from 0% to 7.4%, while amino acid divergence ranged from 0% to 4.6%. Phylogenetic analysis indicated traffic of virus variants through large geographic areas and suggested that migration of infected people may be an important mechanism of virus dispersal. Isolation of vaccine virus from a patient with a fatal case suggests that vaccine-related illness may have been misdiagnosed in the past.

## Abstract 2

Examining viral isolates collected over 66 years shows divergence into clades and potential dispersal by human migration.

Yellow fever virus (YFV) is transmitted by the bite of infected mosquitoes and produces a severe hemorrhagic fever in humans. Despite a safe and effective vaccine (17D), YFV continues to be a public health problem in tropical areas of Africa and South America (*1**–**3*). A recent upsurge in YFV activity in Brazil and the reinfestation of urban areas with the vector mosquito *Aedes aegypti* have stretched disease surveillance and control resources to their limits. During 2000, YFV occurred near Brasilia, the capital city (*4*); in 2001, YFV spread to new areas of Minas Gerais outside the currently recognized enzootic zone (*5*).

In South America, YFV is maintained in enzootic cycles involving monkeys and forest-canopy mosquitoes of the genera *Haemagogus* and *Sabethes* (*6**,**7*). In Brazil, three geographic zones have been defined where YFV circulates (*7*) (Figure 1): 1) the region of endemicity, in which the virus is maintained in mobile monkey populations and where human cases are sporadic and rare; 2) transitional zones of emergence, in which contact is frequent between monkey and human populations (and infected mosquito vectors); and 3) regions of epidemicity where the density of susceptible human populations and competent vector species are both high, and the potential for explosive urban outbreaks is great. Brazil currently has a population of about 176 million; approximately 30 million people live in the YFV-endemic zone, 18 million live in the zone of emergence, and 128 million reside along the Atlantic coast in the YFV-free zone (*8**,**9*).

**Figure 1:**
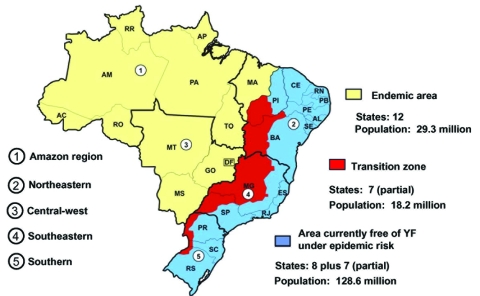
Regions where yellow fever is endemic in Brazil.

Many aspects of the molecular epidemiology and transmission cycles of YFV in the forests of South America are poorly understood, and previous studies of YFV in South America were limited to a relatively small number of isolates. We examined the genetic diversity of 79 YFV strains isolated from Brazil over 67 years and mapped the distribution of variants to investigate patterns of virus divergence and dispersal.

## Material and Methods

### Study Area

Brazil is a country of enormous size and diversity, covering 8,512,000 km^2^. The country is divided into 26 states and the Federal District. These make up five major geographic regions, characterized by broadly different climate and vegetation zones and a highly variable distribution of the human population. The five regions (Figure 1) consist of the northern Amazon region, northeastern region (*caatinga*, very dry), central-western region (swamp and savannah), southeastern region (most heavily populated with an extensive system of roads and railways), and the more temperate southern region bordering Argentina and Paraguay (*10*).

### Virus Strains

Strain-specific data such as geographic locality, passage history, source of isolate, and clinical outcome (for selected human cases) for the 79 Brazilian YFV strains used in this study are provided in Table A1. All strains were originally isolated in suckling mice and cultured once in C6/36 cells to produce seed stocks (Table A1) at the Instituto Evandro Chagas (IEC), Belém, Brazil. Methods for cell culture and virus growth have been previously described (*11**–**13*), and standard laboratory precautions within biosafety level 3 facilities were undertaken to prevent cross-contamination of strains. Thirty-eight (48%) virus strains were from humans (24 from patients who died); 7 (9%) were from monkeys; and 34 (43%) were from mosquito pools, mainly *Haemagogus janthinomys*. Viruses represented a period of 67 years, but with unequal sample distribution: 15% of strains were obtained from 1935 to 1969, 43% were obtained from 1970 to 1989, and 42% were obtained from 1990 to 2001. The year of virus isolation is indicated in the sequence identification (e.g., Brazil35, isolated in 1935). Isolates were from 12 states: Amapá (n = 1), Bahia (n = 1), Federal District (n = 1), Goiás (n = 10), Maranhão (n = 6), Minas Gerais (n = 7), Mato Grosso (n = 4), Mato Grosso do Sul (n = 5), Pará (n = 39), Rondônia (n = 2), Roraima (n = 1), and Tocantins (n = 2).

### Phylogenetic Analysis

Supernatants of YFV-infected Vero cells were obtained, and viral RNA was extracted by using a commercial kit (Qiagen, Valencia, CA) and processed according to the manufacturer's instructions. RNA obtained was stored at –70°C. The genomic-sense degenerate primer EMF (5´ TGGATGACSACKGARGAYAT) and genomic-complementary primer VD8 (5´ GGGTCTCCTCTAACCTCTAG) were used for reverse transcription–polymerase chain reaction amplification of a 595-bp fragment comprising 255 nucleotides of NS5 and 340 nucleotides of 3´NCR (*14**,**15*). PCR products were screened by agarose gel electrophoresis. Bands were recovered with a gel extraction kit (Qiagen) and directly sequenced with an ABI automatic sequencer at the University of Texas Medical Branch protein chemistry core facility.

Sequence editing and alignments were performed with Vector NTI (Informax, Frederick, MD), and additional manual editing of alignments was performed with the GCG Wisconsin Package Version 10.3 (Accelrys, San Diego, CA). The PAUP* program (*16*) was used to infer phylogenetic trees by the neighbor-joining method with Kimura 2-parameter distance corrections. Support for individual clades was determined by nonparametric bootstrapping with 1,000 replicates. The tree was rooted with a sequence of the prototype West African YFV strain Asibi (*17*) and the 17DD substrain vaccine virus (*18*).

## Results

### Genetic Diversity of Brazilian YFV Strains

Sequence data were obtained for a genomic region spanning the terminal portion of NS5 and proximal region of the 3´NCR for 54 Brazilian YFV strains. In addition to the sequence data generated in this study, partial or complete 3´NCR sequences were available for an additional 25 Brazilian YFV isolates (*13**,**14*), which yielded the full dataset of 79 YFV strains from 12 states (alignment length, 576 nt). The NS5/3´NCR sequences contained 148 variable sites, of which 89 were informative. Thirty-two of the informative sites (36%) fell within the NS5 coding portion of the sequence, and 57 (64%) fell within the 3´NCR. Amino acid pairwise divergence among the partial NS5 sequences (85 amino acids in length) ranged from 0% to 4.7% (mean 2.2%).

**Figure 2:**
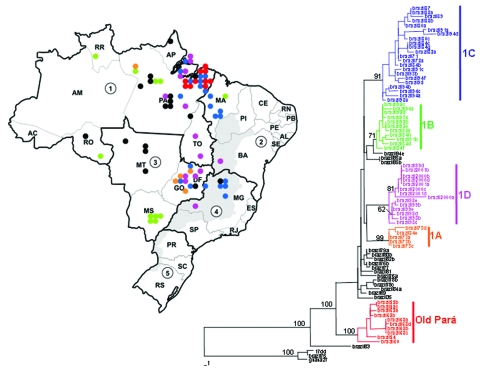
Brazilian NS5/3´NCR phylogeny (576 nt) based on yellow fever isolates (neighbor-joining tree, Kimura 2-parameter distance correction, midpoint rooted). Geographic origin of isolates is indicated on map. 1: North (AC, Acre; AM, Amazonas; AP, Amapá; PA, Pará; RO, Rondônia; RR, Roraima; TO, Tocantins). 2: Northeast (AL, Alagoas; BA, Bahia; CE, Ceará; MA, Maranhão; PB, Paraiba; PE, Pernambuco; PI, Piaui; RN, Rio Grande do Norte; SE, Sergipe). 3: Central West (DF, Distrito Federal; GO, Goiás; MT,Mato Grosso; MS, Mato Grosso do Sul). 4: Southeast (ES, Espírito Santo; MG, Minas Gerais; RJ, Rio de Janeiro; SP, São Paulo). 5: South (PR, Paraná; SC, Santa Catarina; RS, Rio Grande do Sul). Colors correspond to genetic clade structure. Black dots refer to isolates with unresolved phylogenetic position.

A phylogenetic tree of the 79 Brazilian YFV sequences is shown in Figure 2. The tree is rooted with the homologous region of the Asibi prototype strain (parental virus to the 17D vaccine), isolated in Ghana in 1927 (*17*). Mosquito- and vertebrate-derived sequences were distributed randomly throughout the phylogenetic tree. With the exception of one 1983 isolate from Rondônia (BeH 413820) and one 1975 isolate from Aripuana in Mato Grosso (BeH 291597), all Brazilian YFV strains formed a single monophyletic clade. Two major subclades were evident: a subclade comprising isolates from Pará dating from 1954 to1968 and a subclade containing all remaining isolates from 1969 to 2001.

### Variation within the Dominant Subclade

Genetic variation within the dominant subclade of Brazilian YFV strains showed a complex pattern of relationships that demonstrated both geographic and temporal associations. Varying levels of bootstrap support were evident for four clusters (groups 1A–1D) (Figure 2): group 1A included isolates from the 1972–1973 outbreak in Goiás and a 1984 isolate from Pará, group 1B represented sporadic human cases and samples obtained during routine surveillance in western and central regions from 1984 to 1996, group 1C consisted of isolates from sporadic cases and surveillance in eastern central states from 1971 to 1998, and group 1D represented epizootic activity from 1998 to 2001. The phylogenetic position of 12 additional isolates from Pará (10), Amapá (1), and Mato (1) was not easily resolved (Figure 2), so relationships among these strains require further sequence analysis.

The four isolates from the 1972–1973 epidemic in Goiás (group 1A, Brazil73a, 73b, 73c, and 73d) differed by 2–3 nt (0.34%–0.5%) over the length of the NS5/3´NCR fragment (595 nt). One isolate obtained 11 years later (Brazil84e) from a pool of *Haemagogus janthinomys* mosquitoes collected in far northern Pará (Faro), was nearly identical to the 1973 Goiás strains (2–3 nt difference).

Group 1B consisted of 11 isolates. These included two isolates from patients who died (Brazil84h and Brazil91b), isolates from patients in Mato Grosso do Sul (Brazil92c) and Maranhão (Brazil93a), isolates from *Haemagogus* mosquitoes in western Pará (Brazil84d) and Rondônia (Brazil96a), and four isolates obtained in 1992 from mosquito pools (Brazil92a, 92b, 92d, and 92e). Although most strains in group 1B were identified in western and central regions (over a distance as large as 3,500 km^2^), one isolate identified in this cluster was from the northeast region in Barra do Corda, Maranhão (Brazil93a). Isolates collected from contemporaneous *Haemagogus* and *Sabethes* mosquito pools (Brazil92a, 92b, 92d, and 92e) were 100% identical.

Group 1C formed the largest cluster, with 22 strains diverging by 0% to 3.8% (0–27 nt, bootstrap 80%). These variants were distributed during an extended period from 1971 to 1994 throughout the northern Amazon region (Pará: Brazil87, 91a, 84c, 84g, 71, 78a, 84b, 94c, and 98a; Maranhão: Brazil80c, 82a, 93b, 94f, and 95), in central Goiás (Brazil88a, 80a, and 91c), and near the edge of the enzootic zone in Minas Gerais (Brazil89, 88b, 94d, 94b, and 94a). A pair of Minas Gerais isolates (Brazil94b and 94a) obtained from *Haemagogus* and *Sabethes* mosquito pools showed a 100% match across the length of the sequence fragment.

With one exception (Brazil98a), all isolates collected during 1998 to 2001 fell within a single cluster (group 1D) that had 0%–2.7% (0–16 nt) divergence. Although group 1D consistently clustered on neighbor-joining and parsimony trees, bootstrap support was low (53%). The distribution of samples collected during this period closely reflected the southward dispersal of epizootic activity. In 1998, a large number of YF cases occurred in northern Brazil, especially in Pará. In 1999 through 2000, a few cases were reported in Bahia and Tocantins, but the largest number were in central Goiás (*19*). In 2001, YF cases were detected in the transitional zone of Minas Gerais and further east. The isolate from Tocantins collected in 2000 (Brazil2000a) was characterized by an exceptionally large number of substitutions in the 3´NCR region. A single isolate (Brazil98a) collected from a howler monkey in Afua, Pará during the early period of the epizootic did not cluster with the other group 1D strains.

### Subclade of Older Pará Strains

A group of ten isolates from Pará dating from 1954 through 1968 differed from all other Brazilian strains by 5.7% ± 1.6%. These results confirmed previous observations based on analysis of prM/E gene sequences (*14*), indicating 7.8% ± 2.0% divergence for this group of older Pará isolates. To date, 29 YFV strains from Pará dating from 1969 through 1999 have been examined; none of these more recent strains cluster with the earlier clade. Sequence divergence of the older Pará strains approached the threshold level used to define separate YFV genotypes (8%–9%) (*20*).

**Figure 3:**
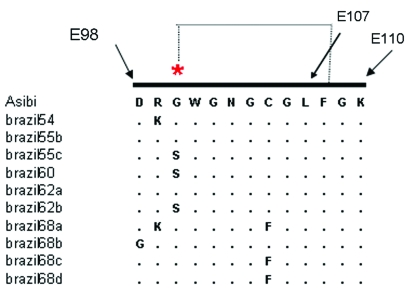
Sequence alignment of the fusion peptide of the envelope (E) gene of selected yellow fever virus (YFV) strains (E98–E110). The Asibi prototype strain indicates the conserved sequence present in the majority of YFV strains and other mosquitoborne flaviviruses. A salt bridge between residues Asp E98 and Lys E110 generates the "CD loop" of residues E100–E108 (*21*).

Alignment of previously published sequences for the structural gene region (223 codons of prM/E) (*14*) showed that 8 of the 10 older Pará strains had one or more mutations within the fusion peptide of the E protein (Figure 3) that is highly conserved among all flaviviruses; it is located in domain II (E98–E110) and plays a role in mediating the acid-catalyzed fusion of virions with target cell membranes (*22*). Three strains from 1968 (Brazil68a, 68c, and 68d) showed a C→F substitution at E105, eliminating one of the disulfide bonds that forms the structural architecture of domain II (*22*). Brazil68b had a D→G mutation at E98, and Brazil54 and Brazil68a shared an R→K mutation at E99. Three of the strains (Brazil55c, Brazil60, Brazil62b) had a G→S substitution at E100. The functional importance of these mutations is unknown. Of the older Pará strains, only Brazil55b and Brazil62a had the consensus sequence for the fusion peptide (Figure 3). In addition, 12 non-Pará subclade strains exhibited substitutions in the fusion peptide sequence.

Preliminary phenotypic differences among three of the older Pará strains (Brazil55b, Brazil60, and Brazil68c) in the standard mouse neuroinvasiveness model (i.e., intraperitoneal injection of 8-day-old suckling mice) (*23*) indicated that the strains with substitutions in the fusion peptide were less lethal (average lethal dose-50 [LD_50_] for Brazil60 and Brazil68c = 5.5 log_10_ tissue culture infective dose-50 [TCID_50_]) than the strain with the consensus sequence (Brazil55b) (LD_50_ = 0.1 log_10_ TCID_50_). Average survival times of the infected mice were 9.2, 11, and 8.8 days, respectively, for the three strains. All three strains achieved titers 6–7 log_10_ TCID_50_/mL when grown in Vero cell culture. Brazil55b had a longer passage history than other YFV strains (4 passages in suckling mouse brain) (Table A1); thus the altered phenotype may be partly attributed to selection during repeated mouse passage.

### Additional Brazilian Isolates

Two Brazilian strains (Brazil83 from Rondônia and Brazil75 from Mato Grosso) failed to group within either of the two major subclades. Brazil83 appears to represent South American genotype II, as it matched 97.2% to the homologous NS5/3´NCR region of Peruvian YFV strains (*14*) and diverged from other Brazilian strains by 6.2% to 13.8% (mean 10.2%). Brazil75 was a vaccine virus, as it matched the 17DD vaccine virus by 99.8%. Sequence comparison of 17DD with Brazil75 revealed a single mutation (C→T) at position 16 of the 3´NCR (*13*).

## Discussion

**Figure 4:**
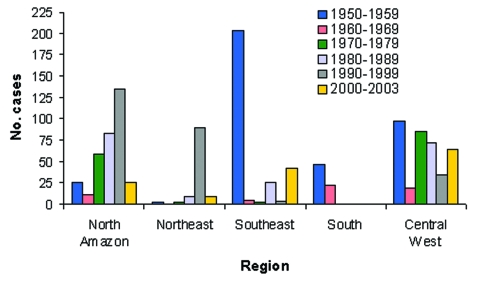
Yellow fever incidence in Brazil by region, 1950–2003.

Brazil accounts for approximately 25% of all YF cases reported from South America (4). YFV activity has been reported from each of the five regions in the country (*2**,**24*). From 1950 to 2003, no single region consistently produced the highest number of YF cases; however, YFV activity in the dry northeast was rare (Figure 4). Overall, the largest number of cases was reported from Goiás (central-western) and Pará (northern) regions.

Previous studies of the genetic relationships among global variants of YFV have indicated that divergence across the length of the ≈11-kb genome is relatively uniform, and the 3´NCR contains useful markers for subtype-specific distinctions (*13**,**25*). Our data showed a single genotype of YFV circulating in Brazil (South American genotype I), with the exception of a single strain from Rondônia (South America genotype II). The Brazilian strains made up two subclades: a group of older strains from Pará from 1954 to 1968 and a larger group dating from 1969 to the present. The clear genetic distinction between strains isolated before 1968 (10 in total) and all subsequent strains suggests that the older Pará strains have been replaced by a dominant new lineage. Several members of the older Pará subclade had one or more mutations within the fusion peptide region of the E protein, including substitutions that disrupted conserved disulfide bridges (Figure 3). Although the functional importance of these substitutions is unknown, changes in the fusion peptide would affect protein folding and conformation and binding interactions with target membranes (*22*). Continued sampling and surveillance of YFV strains from Pará are necessary to confirm whether the variants represented in this subclade have truly become extinct or whether they are being maintained in cycles that have yet to be identified.

At least three articles in the past 50 years have reported YFV epizootics that began in northern and western Amazonian regions and then spread south into Paraná, Santa Catarina, and Rio Grande do Sul. The first was a large epidemic that swept south from Goiás in the 1930s and 1940s (*26*). The second was in 1963, when an epizootic started in Mato Grosso and extended eventually as far south as Missiones and Corrientes Provinces in northern Argentina by 1966 (*27*). In 1972 to 1973, an outbreak occurred in Goiás, and although investigations at the time suggested the epizootic was highly localized (*28*), cases reported from Paraguay in the following years were attributed to spread of viruses from Goiás. Virus maintenance and dispersal have presumably involved sequential infections of migrating groups of monkeys (*29**–**32*). Four isolates collected during the 1972–1973 Goiás outbreak were included in this study; these isolates (group 1A, Brazil73a–d) had nearly identical (99.6%) NS5/3´NCR sequences, and a fifth isolate with a highly similar sequence was obtained 10 years later from Faro, in northern Pará. Other than the single Faro isolate (Brazil84e), no additional descendents have been identified of those outbreak strains.

YFV transmission was particularly active during the rainy season in Maranhão in 1993 to 1994 (*33*). Serologic studies of rural and urban populations at the time indicated an overall attack rate of 75 per 1,000; incidence of clinical disease was 3.5 per 1,000 persons in urban areas and 4.2 per 1,000 in rural areas. Five of the six Maranhão isolates from this time were in group 1C (Figure 2), whereas one isolate from a patient with a fatal case (Brazil93a) appeared to be related to group 1B (Figure 2). In addition to the 1993–1994 outbreak in Maranhão, the subclade of group 1C strains was also associated with sporadic cases throughout 1971 to 1998 in Pará, Minas Gerais, and Goiás.

The most recent increase in epizootic YFV activity in Brazil occurred from 1998 to 2001, with cases distributed over a large region covering eight states (*4*). Several human cases were reported to have originated from the Chapada dos Veadeiros National Park, a tourist canyon located near Brasilia, Goiás. The proximity to major cities raised alarm, and reports at the time designated Goiás as the epicenter of the outbreak (*4*). The phylogenetic evidence presented here indicates that YFV activity in 1998 in Pará, in 2000 in Goiás, and in 2001 in Minas Gerais was all part of one continuous epizootic, which dispersed genetic variants from the 1D group of viruses (Figure 2).

Investigations in 2000 and 2001 indicated that YFV activity had expanded beyond the typical borders of the enzootic zone, into areas where the virus had not been reported for >100 years. The appearance of nearly identical variants across very large distances over short periods (e.g., group 1D spanning >3,000 km within 1 month, group 1B strains with isolates >2,000 km apart within 1 year) suggests that humans, rather than other primates or mosquitoes, may be partly responsible for the spread of YFV variants. Because *Haemagogus* mosquitoes are forest species and are relatively fragile, patterns of traffic and commerce would not likely lead to translocation of infected mosquitoes. From 1998 to 2001, the virus may have been transported from Pará (*34*) to Goiás> (*4*) and then to Minas Gerais by asymptomatic carriers or viremic persons in the prodromal phase. The general trend in the southward movement of the virus (group 1D, Figure 2) could be interpreted to reflect the pattern of labor migration from less populated areas of the northern Amazon to cities of the southeast. However, humans are not believed to play a major role in either virus maintenance or dispersal within South America, because human contact with the forest species *Haemagogus janthinomys* is infrequent, and the length of the viremic period in infected humans is brief (3–4 days).

The identification of one strain from western Rondônia (Brazil83) with high identity to South American genotype II strains (from Peru and Bolivia) suggests that the two South American YFV genotypes cocirculate in regions of western Brazil. The genetic divergence and distribution of YFV are similar that of Oropouche virus (an *Orthobunyavirus* transmitted by midges). Like YFV, Oropouche virus has split into two distinct genotypes in South America with separate eastern and western regions of circulation, and the only region where the two genotypes have been found to cocirculate is in western Brazil, in Rondônia (*35*). The ecologic correlates for these patterns remain uncertain; however, they suggest that surveys of the border regions between Brazil, Peru, and Bolivia may identify additional YFV genotypes.

One unanticipated result of this study was identifying a vaccine virus (Brazil75) from what had been presumed to be a case of natural exposure to wild-type YFV. This isolate was obtained from the blood of a patient who died in Aripuanã, Mato Grosso; this patient had been vaccinated 5 days before becoming ill and died 9 days postvaccination. Serious adverse effects resembling wild-type YF (viscerotropic disease) have only recently been reported (*19**,**36**,**37*). The complete genomic sequence of Brazil75 is currently being sought to address the question of vaccine reversion. Although no controlled studies have examined the safety or efficacy of YF vaccination in immunosuppressed patients, these findings underscore the importance of evaluating patient history before delivering vaccine and also suggest that other cases of vaccine-related illness may have been misdiagnosed in the past.

In summary, we describe considerable genetic variability among YFV variants circulating in Brazil and identify clusters of strains associated with epizootics in different geographic regions. Brazilian YFV strains have diverged into two subclades, one of which has become the dominant lineage in recent years. We suggest a potential role for human migration in mediating virus dispersal. Expansion of YFV outside the enzootic zone presents an ongoing risk for reintroduction of the virus to urban areas and highlights the need for continued surveillance and control.

## Appendix

**Table A1:** Virus isolates used in this study

Isolate	Strain ID	Passage level^a^	Source^b^	State^c^	Community	Biotope^d^/Climate^e^
JSS	Brazil35 (*38*)	Mosq1, SM8	Human^unk^	MT	No data	WC/EHW
Be H111	Brazil54 (*39*)	c6/36, SM10	Human *	PA	Oriboca	EA/EHW
BeAr 162	Brazil55B	SM4, C6/36#1	*Hg. janthinomys*	PA	Pirelli Marituba	EA/EHW
BeAr 189	Brazil55C (*39*)	sm1 c6/36#1	*Sabethes* sp.	PA	Pirelli Marituba	EA/EHW
BeAn 23536	Brazil602	SM1 c6/36#1	*Cebus* sp.	PA	Belém Brasília Km94	EA/EHW
BeAr 46299	Brazil62A (*39*)	c6/36	*Haemagogus* sp.	PA	Belém Brasília Km94	EA/EHW
BeAr 44824	Brazil62B (*39*)	SM1 c6/36#1	*Haemagogus* sp.	PA	Belém Brasília km87	EA/EHW
BeAn142028	Brazil68A2	c6/36#1	*Saguinus* sp.	PA	Abaetetuba	EA/EHW
BeH141816	Brazil68B	SM3, c6/36#1	Human^unk^	PA	Abaetetuba	EA/EHW
BeAr142658	Brazil68C (*39*)	SM2, c6/36#1	*Haemagogus* sp.	PA	Barcarena	EA/EHW
BeAn142027	Brazil68D (*39*)	Original	*Saguinus midas*	PA	Abaetetuba	EA/EHW
BeH171995	Brazil69A	SM1 c6/36#1	Human ^+^	PA	Belém	EA/EHW
BeH203410	Brazil71 (*39*)	SM2, c6/36#1	Human^unk^	PA	Peixe Boi	EA/EHW
BeAr233164	Brazil73A (*39*)	Mosq 4	*Hg. janthinomys*	GO	Goiás	WC/TMW
BeAr232869	Brazil73B (*39*)	Mosq 1, SM2	*Haemagogus* sp.	GO	Goiás	WC/TMW
BeAr233436	Brazil73C (*39*)	Original	*Haemagogus* sp.	GO	Bela Vista	WC/TMW
BeH233393	Brazil73D	SM1 c6/36#1	Human^+^	GO	Mara Rosa	WC/TMW
BeH291597	Brazil75	SM2, Mosq 1	Human ^+^	MT	Aripuanã	WC/EHW
BeAr301129	Brazil76A	SM1 c6/36#1	*Haemagogus* sp.	PA	Alenquer	EA/EHW
BeH301035	Brazil76B	SM1 c6/36#1	Human *	PA	Alenquer	EA/EHW
BeH324213	Brazil77	SM1 c6/36#1	Human *	PA	Altamira	EA/EHW
BeH350698	Brazil78A (*38**,**39*)	SM2, Mosq 1	Human ^unk^	PA	Tomé Acu	EA/EHW
BeAr350397	Brazil78B	SM2, Mosq 1	*Haemagogus* sp.	PA	Faz. Cangalha Formosa	EA/EHW
BeH340824	Brazil78C	SM1 c6/36#1	Human ^+^	PA	Belterra-Santarém	EA/EHW
BeH371220	Brazil79A	SM1 c6/36#1	Human ^+^	PA	Altamira	EA/EHW
BeAr378600	Brazil80A	SM2, C6/36#1	*Haemagogus* sp.	GO	Uruacu	WC/TMW
BeH385780	Brazil80B (*39*)	SM1 c6/36#1	Human ^+^	PA	Altamira	EA/EHW
BeH379501	Brazil80C	SM2	Human ^+^	MA	Montes Altos	NEW/EHS
BeH394880	Brazil81	Original, C6/36#1	Human *	PA	Conceição Araguaia	EA/EHW
BeH403366	Brazil82A	SM1 c6/36#1	Human ^+^	MA	Amarante do Maranhão	NEW/EHS
BeH405954	Brazil82B	SM1 c6/36#1	Human *	PA	Altamira	EA/EHW
BeH 413820	Brazil83	SM1 c6/36#1	Human ^+^	RO	Porto Velho	WA/EHW
BeH425381	Brazil84A (*39*)	c6/36	Human ^+^	AP	Tribo Oyampi	NA/EHW
BeAn 424208	Brazil84B	Original, C6/36#1	*Chiropotes satanas*	PA	Tucuruí	EA/EHW
BeAr424719	Brazil84C	SM1 c6/36#1	Haemagogus *sp.*	PA	S. Domingos	EA/EHW
BeAr424083	Brazil84D	SM1 c6/36#1	*Hg. albomaculatus*	PA	Monte Alegre	EA/EHW
BeAr424492	Brazil84E	SM1 c6/36#1	*Hg. janthinomys*	PA	Faro	EA/EHW
BeH422255	Brazil84F	SM1 c6/36#1	Human ^+^	PA	Faro	EA/EHW
BeH423602	Brazil84G	SM1 c6/36#1	Human ^+^	PA	S. Domingos	EA/EHW
BeH422312	Brazil84H	SM1	Human ^+^	PA	Monte Alegre	EA/EHW
BeH436823	Brazil85A	SM1 c6/36#1	Human ^+^	MT	Sinop	WC/EHW
BeAr437159	Brazil85B	SM1 c6/36#1	*Hg. janthinomys*	MT	Sinop	WC/EHW
BeH463676	Brazil87	SM1 c6/36#1	Human ^+^	PA	Breves	EA/EHW
BeH474245	Brazil88A	SM1 c6/36#1	Human ^+^	GO	Caiapônia	WC/TMW
BeH474297	Brazil88B	SM1 c6/36#1	Human ^+^	MG	Paracatu	SE/TSS
BeH485717	Brazil89	SM1 c6/36#1	Human ^+^	MG	Francisco Dumont	SE/TSS
BeAr 511437	Brazil91A2	SM1, C30c6/36#1	*Hg. janthinomys*	PA	Barcarena	EA/EHW
BeH511843	Brazil91B	SM1, C6/36#2	Human ^+^	RR	Tribo Yanomamy	WA/EHS
BeAn 510268	Brazil91C	SM1 c6/36#1	*Alouatta* sp.	GO	Goiás	WC/TMW
BeAr 512943	Brazil92A (*39*)	SM1 C6/36-1 Vero2	*Hg. janthinomys*	MS	Sidrolândia	WC/TMW
BeAr513008	Brazil92B (*39*)	SM1 c6/36#1	*Sabethes* sp.	MS	Sidrolândia	WC/TMW
BeH512772	Brazil92C (*39*)	SM2, C6/36#1	Human ^unk^	MS	Campo Grande	WC/TMW
BeAr513060	Brazil92D	SM1 c6/36#1	*Haemagogus* sp.	MS	Campo Grande	WC/TMW
BeAr 513292	Brazil92E (*39*)	SM1 C6/36-1 Vero1	*Sabethes cloropterus*	MS	Jaraguarí	WC/TMW
BeH520988	Brazil93A	SM1 c6/36#1	Human ^+^	MA	Barra do Corda	NEW/EHS
BeH521244	Brazil93B	SM1 c6/36#1	Human *	MA	Mirador	NEW/EHS
BeAr 527785	Brazil94A (*39*)	SM1 c6/36#1	*Sabethes cloropterus*	MG	Arinos	SE/TSS
BeAr527198	Brazil94B (*39*)	SM1 c6/36#1	*Haemagogus* sp.	MG	Arinos	SE/TSS
BeAr527547	Brazil94C	SM2, vero1	*Haemagogus* sp.	PA	Don Eliseu	EA/EHW
BeH526722	Brazil94D	SM2, c6/36#1	Human ^+^	MG	Arinos	SE/TSS
BeAr528057	Brazil94E	Original, C6/36#1	*Hg. janthinomys*	MG	Arinos	SE/TSS
BeAr527410	Brazil94F	Original, C6/36#1	*Hg. janthinomys*	MA	Pastos Bons	NEW/EHS
BeH535010	Brazil95	SM2, c6/36#1	Human ^+^	MA	Pastos Bons	NEW/EHS
BeAr 544276	Brazil96A (*39*)	SM1 c6/36#1	*Hg. janthinomys*	RO	Cabixi	WA/EHW
BeAn 604552	Brazil98A	Original, C6/36#1	*Alouatta belzebul*	PA	Afua	NA/EHW
BeAr603401	Brazil98B	Original, C6/36#1	*Hg. janthinomys*	PA	Altamira	EA/EHW
BeAr605158	Brazil98C	SM1 c6/36#1	*Hg. janthinomys*	PA	Afuá	NA/EHW
BeH603325	Brazil98D	Original, C6/36#1	Human ^unk^	PA	Afuá	EA/EHW
BeH605427	Brazil98E	SM1 c6/36#1	Human ^+^	PA	Carajas	EA/EHW
BeAr614320	Brazil99B	SM2	*Haemagogus* sp.	PA	Breves	NA/EHW
BeAr617127	Brazil99D	SM1 c6/36#1	*Hg. janthinomys*	TO	Monte do Carmo	EA/EHS
BeH613582	Brazil99E	SM1 c6/36#1	Human ^*^	PA	Breves	NA/EHW
BeAr628124	Brazil00A (*39*)	SM1 c6/36#1	*Hg. janthinomys*	TO	Paranã	EA/EHS
BeH 622491	Brazil00B	SM1	Human ^+^	DF	Brasilia	WC/THA
BeAn625923	Brazil00C	Original, C6/36#1	*Alouatta* sp.	GO	Goiás	WC/TMW
BeH 622205	Brazil0D	SM1 c6/36#1	Human ^*^	GO	Goiás	WC/TMW
BeAr630768	Brazil01A	SM1	*Hg. janthinomys*	GO	Alto Paraiso	WC/TMW
BeAr631464	Brazil01B	SM1 c6/36#1	*Sa. chloropterus*	BA	Jaborandi	NED/THA
BeAr645693	Brazil01D	SM1 c6/36#1	*Haemagogus*	MG	Uberaba	SE/TSS

^a^Passage history of seed strain in collection. SM, suckling mouse. ^b^Outcome of human cases, unk = unknown, ^+^ = fatal, * = recovered. ^c^AP, Amapa; BA, Bahia; DF, Federal District; GO, Goiás; MA, Maranhão; MG, Minas Gerais; MS, Mato Grosso do Sul; MT, Mato Grosso; PA, Pará; RO, Rondônia; RR, Roraima; TO, Tocantins. ^d^EA, East Amazon; NA, Northern Amazon; NED, Northeast Dry; NEW, Northeast Wet; S, South; SE, Southeast; WA, West Amazon; WC, West-Central. ^e^EHW, equatorial, hot and wet; EHS, equatorial, hot and semi-wet; THA, tropical, hot and semi-arid; THW, tropical, semi-hot and wet; TMW, tropical, mesothermal, wet; TSW, tropical, semi-hot, wet; TSS, tropical, semi-hot and semi-wet.
